# Podocytes derived from human induced pluripotent stem cells: characterization, comparison, and modeling of diabetic kidney disease

**DOI:** 10.1186/s13287-022-03040-6

**Published:** 2022-07-26

**Authors:** Julie Bejoy, Justin M. Farry, Jennifer L. Peek, Mariana C. Cabatu, Felisha M. Williams, Richard C. Welch, Eddie S. Qian, Lauren E. Woodard

**Affiliations:** 1 Division of Nephrology and Hypertension, Department of Medicine, Vanderbilt University Medical Center, Nashville, TN 37232 USA; 2 Department of Biomedical Engineering, Vanderbilt University, Nashville, TN 37232 USA; 3 Department of Veterans Affairs, Tennessee Valley Healthcare System, Nashville, TN 37212 USA

**Keywords:** iPSC, Podocytes, Diabetes, Kidney

## Abstract

**Background:**

In diabetic kidney disease, high glucose damages specialized cells called podocytes that filter blood in the glomerulus. In vitro culture of podocytes is crucial for modeling of diabetic nephropathy and genetic podocytopathies and to complement animal studies. Recently, several methods have been published to derive podocytes from human-induced pluripotent stem cells (iPSCs) by directed differentiation. However, these methods have major variations in media composition and have not been compared.

**Methods:**

We characterized our accelerated protocol by guiding the cells through differentiation with four different medias into MIXL1+ primitive streak cells with Activin A and CHIR for Wnt activation, intermediate mesoderm PAX8+ cells via increasing the CHIR concentration, nephron progenitors with FGF9 and Heparin for stabilization, and finally into differentiated podocytes with Activin A, BMP-7, VEGF, reduced CHIR, and retinoic acid. The podocyte morphology was characterized by scanning and transmission electron microscopy and by flow cytometry analysis for podocyte markers. To confirm cellular identity and niche localization, we performed cell recombination assays combining iPSC-podocytes with dissociated mouse embryonic kidney cells. Finally, to test iPSC-derived podocytes for the modeling of diabetic kidney disease, human podocytes were exposed to high glucose.

**Results:**

Podocyte markers were expressed at similar or higher levels for our accelerated protocol as compared to previously published protocols that require longer periods of tissue culture. We confirmed that the human podocytes derived from induced pluripotent stem cells in twelve days integrated into murine glomerular structures formed following seven days of culture of cellular recombinations. We found that the high glucose-treated human podocytes displayed actin rearrangement, increased cytotoxicity, and decreased viability.

**Conclusions:**

We found that our accelerated 12-day method for the differentiation of podocytes from human-induced pluripotent stem cells yields podocytes with comparable marker expression to longer podocytes. We also demonstrated that podocytes created with this protocol have typical morphology by electron microscopy. The podocytes have utility for diabetes modeling as evidenced by lower viability and increased cytotoxicity when treated with high glucose. We found that multiple, diverse methods may be utilized to create iPSC-podocytes, but closely mimicking developmental cues shortened the time frame required for differentiation.

**Supplementary Information:**

The online version contains supplementary material available at 10.1186/s13287-022-03040-6.

## Background

The discovery of adult human cells that can be reprogrammed into induced pluripotent stem cells (iPSCs) has opened up a new way to generate different cell types in vitro [1]. iPSCs have an unlimited capacity for self-renewal and the ability to develop into most cell types. Many techniques for producing renal cells from iPSCs have been devised based on knowledge gathered from embryonic kidney development [2–4]. Kidneys are formed from the mesoderm, which develops into the metanephros, from which the ureteric bud and metanephric mesenchyme emerge [5]. The collecting duct, renal pelvis, and ureters develop from the ureteric bud, whereas the renal tubules and glomeruli develop from the metanephric mesenchyme [6]. To generate renal cells from iPSCs, most methods rely upon exploiting natural signaling pathways that follow these developmental phases. iPSCs have also been used to create “kidney-in-a-dish” organoids that mimic the growing embryonic kidney [4, 7–9]. However, kidney organoids resemble a human fetal kidney rather than an adult kidney, both morphologically and transcriptionally [9]. Human kidney organoids contain podocytes, but the high level of cellular heterogeneity makes it difficult to determine the target cell type of an insult [10, 11]. Derived kidney organoids contain 10–20% off-target cells such as neurons. Kidney organoid protocols produce a highly variable number of podocytes, with estimates ranging from 4 to 28% [10].

Several methods for producing monocultures of podocytes from iPSCs have been published throughout the last decade [12–17]. Other available methods require a lengthy culture time or expensive medium components, but we recently published a detailed method that required less culture time and lower-cost components [14]. Despite a proliferation of protocols in recent years, the comparative quality and marker expression of iPSC-derived podocytes obtained from each method was unknown. In this study, we compare four methods for the differentiation of podocytes from iPSCs, including our method [14]. In addition, we explore the applications of iPSC-derived podocytes in in vitro systems to study podocyte disease, specifically diabetic kidney disease.

Glomerular diseases are linked to changes in the phenotype of proliferating podocytes. Podocytes are highly differentiated cells that attach to capillaries, forming an important part of the nephron’s glomerular filtration barrier (GFB) [18]. These specialized pericytes have foot-like extensions called foot processes joined by slit diaphragms [19]. Podocyte dysfunction contributes to proteinuria through dedifferentiation, podocyte apoptosis, proliferation arrest, and foot process effacement [20]. As glomerular diseases progress, loss of the podocyte foot process is accompanied by loss of podocyte markers such as Podocalyxin (PODXL) and Synaptopodin (SYNPO). Podocyte loss may be caused by a genetic mutation [21], diabetic nephropathy [22], or nephrotoxic compounds [23]. Because fully differentiated podocytes have limited proliferative capacity, their loss or injury causes GFB leakage leading to proteinuria and end-stage renal disease [24–26].

During kidney development, the crescent-shaped epithelial cells beneath the growing glomerulus differentiate into podocytes [27]. Podocyte precursors are polygonal cells that divide quickly and are joined by apical connections. At this stage, the cells express the tight junction protein Zonula occludens-1 (ZO-1) and the early podocyte marker PODXL [28]. ZO-1 migrates from the apical to the basal region as podocytes differentiate, and the foot process and slit membrane develop [28], expressing slit membrane-associated proteins such as Nephrin (NPHS1) [29], Podocin (NPHS2), and CD2-Associated Protein. Macromolecules are filtered through the slit diaphragm, which is controlled by tight junction proteins [30]. During differentiation, several podocyte marker proteins, including SYNPO [31] and PODXL [32], increase in expression.

As animal models do not perfectly mimic human disease phenotypes [33], methods for producing human podocytes in vitro provide an important approach for uncovering the mechanism of various podocytopathies. In vitro culture of podocytes was introduced in the mid-1970s using cells taken from the renal cortex [34, 35]. Podocytes were extracted by sieving the cultured outgrowth of the isolated glomerulus [36] or by digesting the entire glomerulus [37], but rapid dedifferentiation of cells in vitro was a key drawback of this approach. The shape of these podocytes changed from arborized to cobblestone, indicating a reversion to an immature podocyte phenotype [38]. The only option to maintain the adult phenotype was to isolate fresh cells regularly. To increase proliferative capacity, exogenous human telomerase reverse transcriptase or SV40 big T antigen can immortalize primary cells [39, 40], but this causes their function to be compromised and their morphology to be immature. Although iPSC-derived podocytes have many advantages for modeling of podocytopathies, their wide adoption has been hindered by the absence of a comparison study to determine the similarities and differences between diverse protocols that all produce cells with podocyte markers. In this study, we compared podocytes derived from iPSC and explored their niche specification and utility for disease modeling.

## Methods

### Differentiation of podocytes from iPSCs

#### Ciampi protocol

We used the established protocol to derive the podocytes [17]. iPSCs were grown on Matrigel-coated (Fisher Scientific, Hampton, NH) dishes at a density of 30,000–50,000 cells/cm^2^ in mTesR medium (STEMCELL Technologies, Cambridge, MA) with 10 μM Rho-associated kinase inhibitor Y-27632 dihydrochloride (ROCKi) (STEMCELL Technologies) for 24 h. On day 1, cells were treated with a stage I medium comprised of a 1:1 mixture of Dulbecco’s Modified Eagle Medium/Nutrient mixture F12 (DMEM/F12) plus GlutaMax (Thermo Fisher Scientific, Waltham, MA) and neurobasal media (Thermo Fisher Scientific) with Neuro-2 (N2; Thermo Fisher Scientific) and 1 × B27 (Thermo Fisher Scientific), supplemented with 1 μM CHIR99021 (Reagents Direct, Encinitas, CA) instead of 1 μM CP21R7 and 25 ng/ml bone morphogenetic protein 4 (BMP4; R&D Systems, Minneapolis, MN). At day 4, the cells were treated with stage II STEMdiff Albumin Polyvinyl Alcohol Essential Lipids (APEL) medium (STEMCELL Technologies) in the presence of growth factors including 100 nM retinoic acid (RA; STEMCELL Technologies), 50 ng/ml BMP7 (R&D Systems), and 200 ng/ml fibroblast growth factor 9 (FGF9; Peprotech, Cranbury, NJ) for 2 days. On day 6, cells were dissociated using Accutase (Thermo Fisher Scientific) and 20,000/40,000 cells/cm^2^ were plated on type I collagen-coated plates (Thermo Fisher Scientific). The cells were treated for 7 more days with stage III VRAD podocyte-maintaining medium (DMEM/F12 plus GlutaMax, 10% fetal bovine serum (FBS) (Life Technologies, Carlsbad, CA), 80 μM RA (STEMCELL Technologies), and 100 nM Vitamin D3 (Thermo Fisher Scientific).

#### Rauch protocol

We used the established protocol with optimization to derive the podocytes [41]. iPSCs were seeded onto Geltrex-coated (Fisher Scientific) plates at a density of 9000 cells/cm^2^ and cultured in differentiation medium (medium M1) for 24 h. On day 0, cells received medium consisting of DMEM/Ham F12 (Thermo Fisher Scientific) with 1.25% FBS, 100 μM non-essential amino acids (NEAA) (Thermo Fisher Scientific), and penicillin/streptomycin (P/S) (Thermo Fisher Scientific) with 5 µM ROCKi. Then, from day 1 to day 10 the cells were cultured in medium M1 consisting of DMEM/Ham F12, 1.25% FBS, 100 μM NEAA with 10 ng/ml Activin A (Peprotech), 15 ng/ml BMP7, and 100 nM RA. For the following ten days (day 11 to day 20), differentiated podocyte-like cells were maintained using basic differentiation media devoid of differentiation factors (DMEM/F12, 2.5% FBS, 100 μM NEAA, 1xP/S).

#### Musah protocol

The podocytes were generated using the established protocol with slight modification [12]. Briefly, iPSCs grown until 80% confluency were dissociated using Accutase. The cells were then seeded onto laminin-511 (Peprotech) coated plates and cultured in mTesR media with 10 μM ROCKi for the first 24 h. Then, from day 1 to day 3 the cells were treated with Stage I medium, also called mesoderm differentiation medium, consisting of DMEM/F12 with GlutaMax supplemented with 100 ng/ml Activin A, 3 μM CHIR99021, and 1X concentration of B27 serum-free supplement. From day 3 to day 16, the cells were grown in Stage II medium, called intermediate mesoderm induction medium, consisting of DMEM/F12 with GlutaMax supplemented with 100 ng/ml BMP7, 3 μM CHIR99021, and 1X concentration of B27 serum-free supplement. At day 16, the intermediate mesoderm cells were dissociated and replated into a laminin-511-coated plates and podocyte phenotype was induced using Stage III medium. Stage III medium consists of DMEM/F12 with GlutaMax supplemented with 100 ng/ml BMP7, 100 ng/ml Activin A, 50 ng/ml VEGF (Peprotech), 3 μM CHIR99021, 0.1 μM RA, and 1 × B27 serum-free supplement.

#### Bejoy protocol

iPSCs seeded onto Geltrex-coated plates at a density of 100,000 cells/cm^2^ were treated with mTesR media with 10 μM ROCKi for the first 24 h. On day 0, cells were treated with base media (DMEM/F12 with GlutaMax,1XB27) supplemented with primitive streak induction factors including 100 ng/ml Activin A and 3 μM CHIR99021. On day 2, cells were treated with base media including 8 μM CHIR99021 to induce intermediate mesoderm until day 5, followed by treatment with base medium containing 200 ng/ml FGF9 and 1 µg/ml Heparin (Sigma-Aldrich, St. Louis, Missouri). On day 7, to induce the nephron progenitors, the cells were dissociated using Accutase and replated (1:4) onto laminin-511-coated plates. The podocyte phenotype was induced using base media supplemented with 100 ng/ml BMP7, 100 ng/ml Activin A, 50 ng/ml VEGF, 3 μM CHIR99021, and 0.1 μM RA, and cells were assayed on day 12. The detailed methods for deriving the podocytes were carefully described elsewhere [14].

### Immunocytochemistry

The cells were fixed using 4% paraformaldehyde (PFA) (Thermo Fisher Scientific) and permeabilized with 0.1% Triton X 100 (Sigma-Aldrich). The samples were then blocked and stained with primary antibodies either for 4 h at room temperature (RT) or overnight at 4 °C (Table 1). The cells were then washed and incubated with the corresponding secondary antibody (Table 1). The samples were then stained with DAPI and visualized on either the DM6000 fluorescent microscope (Leica Microsystems, Wetzlar, Germany) or the ZOE™ Fluorescent Imager (Bio-Rad Laboratories, Hercules, CA). Fixed cells were stained using phalloidin to visualize filamentous-actin (F-actin) and 4′,6′-Diamidino-2-Phenylindole (DAPI) to stain the nucleus. Fluorescent images were analyzed using ImageJ software.

**Table 1:** Primary antibodies, secondary antibodies, and stains for flow cytometry, immunostaining, and immunoblotting experiments

Cell type	Target	Origin	Isotype	Company	Catalog #	Dilution
*Primary antibodies*						
Undifferentiated	OCT-4	Rat	IgG2b	R&D systems	MAB1759SP	1:200
Primitive streak	MIXL1	Rabbit	IgG	Proteintech	22772-1-AP	1:200
Intermediate mesoderm	PAX8	Rabbit	IgG	Proteintech	10336-1-AP	1:200
Nephron progenitors	CITED1	Mouse	IgG2a	Fisher scientific	89-335-107	1:200
	SIX2	Rabbit	IgG	Proteintech	11562-1-AP	1:200
Podocytes	PODXL	Rabbit	IgG	Proteintech	18150-1-AP	1:200
	NEPHRIN (NPHS1)	Sheep	IgG	R&D systems	AF4269	1:200
	SYNPO	Rabbit	IgG	Abcam	ab224491	1:200
Podocytes (mouse-specific)	NEPHRIN (NPHS1)	Mouse	IgG	R&D systems	AF3159-SP	1:200
Podocytes (human specific)	MAFB	Rabbit	IgG	Abcam	ab223744	1:200

Visualization of	Binds to	Stain	Channel	Company	Catalog #	Dilution
*Stains*						
Cytoskeleton	F-actin	Phalloidin	594	Molecular Probes	A12381	1:100
Nuclei	DNA	DAPI	blue	Millipore Sigma	D9542	1:1000

Species	Fluorophore	Target	Company	Catalog #	Dilution
*Secondary antibodies*					
Goat	Alexa 488	Anti-mouse IgGa	Life technologies	A-21131	1:200
Goat	Alexa 488	Anti-rat IgG	Life technologies	A-11006	1:400
Goat	Alexa 594	Anti-rabbit IgG	Life technologies	A-11037	1:400
Donkey	Alexa 594	Anti-sheep IgG	Life technologies	A-11016	1:400

### Flow cytometry

To evaluate the expression of proteins of interest quantitatively, the cells were harvested by Accutase treatment and analyzed by flow cytometry. Approximately 1 × 10^6^ cells per sample were first fixed with 4% PFA and washed with staining buffer (2% FBS in phosphate-buffered saline (PBS)). The cells were permeabilized with 100% cold methanol, blocked, and incubated with primary antibodies followed by the corresponding secondary antibody. The cells were acquired with BD FACSCanto II flow cytometer (BD Biosciences, Beckton, New Jersey) and analyzed against isotype controls using FlowJo software.

### Western blotting

Cells grown on a monolayer were washed with cold 1 × PBS and scraped in order to be transferred into microfuge tubes kept on ice. The cells were pelleted using a refrigerated microfuge at 500 × g for 5 min and lysed in cold lysis buffer (radioimmunoprecipitation assay (RIPA) buffer (Sigma-Aldrich), 100X protease inhibitor mix (1X PI, Sigma-Aldrich), 20X PhosStop tablets phosphatase inhibitors (Roche, Basel, Switzerland). Samples containing 10 μg total protein were loaded onto 4–12% Bis/Tris gels (Life Technologies) and run at 170 V for approximately 1 h before transferring to nitrocellulose with the iBlot (Thermo Fisher Scientific). After transfer, blots were blocked for 1 h at room temperature with rocking in 10 ml Tris-buffered Saline (TBS)-based Odyssey Blocking Buffer (TOBB; LI-COR Biosciences, Lincoln, NE). The blots were then rinsed in milli-Q H_2_O for 5 min at room temperature with rocking. Then, 5 ml of the primary antibody dilution made in TOBB + 0.2% Tween 20 (Sigma-Aldrich) (TOBBT) was added and the blot was incubated with rocking at 4 °C. The blots were then washed 3 × with 1 × TBS (LI-COR Biosciences) + 0.1% Tween 20 (TBST). Then, 10 ml/blot secondary antibody diluted in TOBBT + 0.01% sodium dodecyl sulfate (KD Medical, Columbia, Maryland) was added to each blot and incubated with rocking for 1 h at RT. Blots were washed again 3 × with TBST, then 2 × with TBS. The blots were scanned using the Licor Odyssey (LI-COR Biosciences) instrument. The expression was quantified using ImageJ software.

### Albumin uptake assay

The functionality of derived podocytes was measured using an albumin uptake assay [16, 41]. Briefly, differentiated podocyte cultures were cultured in serum-free media for 24 h. The next day, cells were rinsed with PBS and then incubated with 50 μg/ml fluorescein isothiocyanate (FITC)-conjugated bovine serum albumin (Thermo Scientific). For the albumin binding assay, the cells were incubated for 1 h at 4 °C. To evaluate binding and endocytosis, cells were kept at 37 °C for 24 h. Brightfield images and FITC images were taken using a ZOE fluorescence microscope (Bio-Rad Laboratories), and merging of the images was used to determine the albumin uptake.

### Transcript measurements

The total RNA of cells at different stages of differentiation was purified using the RNeasy kit by following the manufacturer’s protocol (Qiagen, Germantown, MD). cDNA was synthesized by reverse transcription of 1 μg of each RNA sample with the iScript cDNA synthesis kit (Bio-Rad Laboratories). Primers specific for target genes (Table 2) were purchased commercially (Real Time Primers, LLC, Melrose Park, PA). The gene glyceraldehyde-3-phosphate dehydrogenase (GAPDH) was used as an endogenous control for normalization of expression levels. Real-time reverse transcription–polymerase chain reaction (RT-PCR) was performed on each sample using SYBR Green PCR Master Mix (Bio-Rad Laboratories). The amplification reactions were performed as follows: 2 min at 50 °C, 10 min at 95 °C, and 40 cycles of 95 °C for 15 s and 55 °C for 30 s, and 68 °C for 30 s. Fold variation in gene expression was quantified by means of the comparative C_t_ method.

**Table 2:** Primer sequences to detect podocyte transcripts by RT-PCR

Primer	Forward sequence	Reverse sequence
SYNPO	GAT GTC AAC CAA AAC CTT GC	GTG CCA TTA GAT GGG AGT TG
PODXL	AGG CTT GAG TGA GGT GTT TG	AGC CTT TGA TTG ATT TGC AG
WT1	TCA TCA CTG GGA GTG TCC TT	TGG ATT TCC TCA CCC AGT AA
GAPDH	CTC TCT GCT CCT CCT GTT CGA	TGA GCG ATG TGG CTC GGC

### MTT assay

The viability of cells in each sample was measured by MTT assay. Briefly, a MTT reaction mixture was prepared and filter sterilized followed by a dilution to 1 mg/ml in HBSS without calcium and magnesium (Fisher Scientific). The monolayer cells were spun at 800 × g for 2 min at room temperature. Then, the media was removed from the wells and 100 μL of 1 mg/ml MTT reagent (Sigma-Aldrich) was added to each well. The solution was gently mixed and incubated at 37 °C in a CO_2_ incubator for 1–2 h. After 2 h, the cells were spun and the MTT supernatant was removed. Next, 100 μL of isopropanol was added to cells and mixed vigorously on an orbital shaker to dissolve the MTT formazan precipitate. The optical density (OD) values were read at 560 and 690 nm. The 690 nm reading was used for background correction.

### Cytotoxicity assay

Cytotoxicity Assay kit G1780 (Promega, Madison, WI) was used to assay the LDH in the media (Promega). Briefly, the cell culture supernatant was collected and spun at 800 × g for 2 min and 50 µL of the supernatant was added to a new 96 well plate. Next, 50 µL/well of CytoTox Assay reagent at RT was added to wells containing media. The plate was incubated in the dark at RT for 30 min. Then, 50 µL 1 M acetic acid stop solution was added to each well and the absorbance was read at 490 nm within 1 h. Average readings from media-only wells were used for blank correction.

### Recombination assay

Cited1-CreER™-GFP transgenic dams pregnant with embryonic day (E) 12.5–15.5 embryos were sacrificed. Two or three embryonic kidneys were dissected, minced, and transferred into Eppendorf tubes for reaggregation. Accutase was used to dissociate the tissues, which were subsequently centrifuged for 5 min to isolate the pellets. The pellet was then mixed well with 50,000 podocytes before being centrifuged again to produce aggregates. The pellet was then transferred to a transwell plate with a polyether sulfone membrane (0.4 µm) and cultured as organoids. The cells were treated with DMEM/F12 medium with 10% Fetal calf serum (FCS) (Thermo Fisher Scientific) and incubated for 7 days at 5% CO_2_, 37 °C. The kidney explants were fixed with 4% PFA for immunostaining on day 7. The fixed samples were stained following the steps of immunocytochemistry mentioned above.

### Statistics

When two groups were compared, we used the Student’s t test. If more than two groups were analyzed, we used the ANOVA with the Bonferroni post-test. We considered p values that are less than 0.05 to be statistically significant.

## Results

### Differentiation of iPSCs to primitive streak, intermediate mesoderm, nephron progenitors, and podocytes

**Fig. 1:**
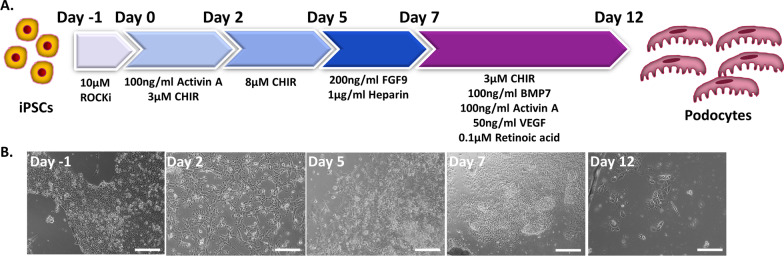
Protocol overview and stages of iPSC-podocyte differentiation. **A** Schematic of the podocyte differentiation protocol from iPSCs. **B** Brightfield images of each stage of differentiation: primitive streak cells (day 2), intermediate mesoderm (day 5), nephron progenitors (day 7), and podocytes (day 12). The derived podocytes have a large cellular body with elongated foot processes. The scale bar is 100 μm

Our accelerated method follows a four-stage process of directed differentiation for converting iPSCs into podocytes [14]. Podocytes were derived from iPSCs via stepwise generation of primitive streak-like cells, intermediate mesoderm cells, proliferative nephron progenitors, and finally podocytes (Fig. 1A).

**Fig. 2:**
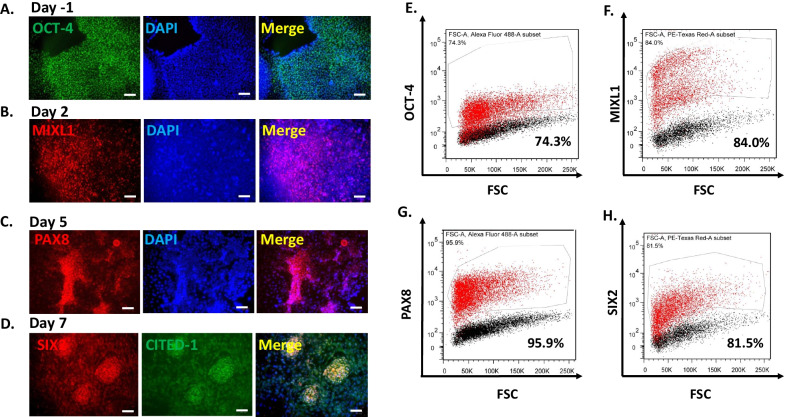
Validation of each stage of podocyte differentiation. Immunostaining of each stage of differentiation showing **A** pluripotency marker octamer-binding transcription factor 4 (OCT-4) at day 1; **B** primitive streak marker MIXL1 at day 2; **C** intermediate mesoderm marker PAX8 at day 5; and **D** nephron progenitor markers SIX2 and CITED1 at day 7. Corresponding flow cytometry analysis of the markers was plotted on the y-axis for **E** OCT-4, **F** MIXL1, **G** PAX8, and **H** SIX2 versus forward scatter (FSC) on the *x*-axis (*n* = 2). Scale bar is 100 μm

We began the differentiation protocol by confirming iPSC pluripotency. The iPSCs possessed a flat, small, and round morphology with well-defined edges (Fig. 1B). iPSCs were also stained for OCT-4 (POU5F1) to verify pluripotency (Fig. 2A). Wingless-related integration site (Wnt) signaling combined with activin signaling has been shown to generate the primitive streak [15, 42]. Therefore, iPSCs were treated for two days with Activin A and the small molecule Wnt signaling activator CHIR99021 (CHIR). Flow cytometry analysis revealed that octamer-binding transcription factor 4 (OCT-4) expression dramatically decreased after initiation of differentiation (Fig. 2E; Additional file 1: Fig. S1A). The resulting cells uniformly expressed primitive streak marker Mix Paired-Like Homeobox (MIXL1) by immunostaining and flow cytometry analysis (Fig. 2B, F). Prolonged activation of Wnt signaling supports iPSC differentiation into the intermediate mesoderm [9]. Primitive streak was treated with a higher concentration of CHIR99021 (8 μM from 3 μM) for 2–3 days to induce the differentiation of PAX8 + intermediate mesoderm cells (Fig. 2C) and reduce the population of MIXL1 + cells to 26.7%. We performed flow cytometry and found that 95.9% of cells expressed PAX8 at day 5 following the differentiation to the intermediate mesoderm stage (Fig. 2G).

Since a high concentration of FGF9 has been demonstrated to generate nephron progenitors from intermediate mesoderm cells [9, 17], we added the same concentration of FGF9 (200 ng/ml) [9] along with heparin (1 µg/ml) to stabilize FGF9. At day 7 of differentiation, the nephron progenitors took on a cobblestone-like appearance and expressed nephron progenitor markers SIX Homeobox 2 (SIX2) and Cbp/p300 interacting transactivator with Glu/Asp rich carboxy-terminal domain 1 (CITED1) by immunostaining (Fig. 2D) and flow cytometry analysis (Fig. 2H). Activation of various cell signaling pathways is required for the generation and maintenance of podocytes during kidney development including BMP7 [17], retinoic acid [43], and vascular endothelial growth factor (VEGF) [44]. We treated nephron progenitors with the Musah podocyte media containing a cocktail of growth factors for 5 days [12]. The resulting iPSC-derived podocytes showed large, arborized morphology at day 12 (Fig. 1B). We repeated our podocyte differentiation protocol on three independently-derived iPSC lines from two genetic backgrounds: DYR0100 (origin: SCRC-1041 foreskin fibroblast cell line), MAFB:mTagBFP2/GATA3mCherry (origin: CRL-2429 foreskin fibroblast cell line), and LRP2:mTagBFP2 (origin: CRL-2429 foreskin fibroblast cell line) [45–48] (Additional file 1: Fig. S2). We found that the CHIR99021 treatment time may need to be adjusted between 2–3 days depending on the cell line used.

**Fig. 3:**
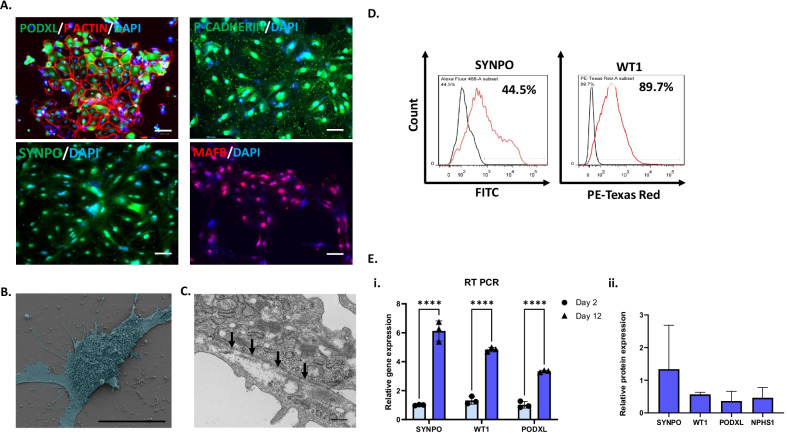
Characterization of human podocytes derived from iPSCs in 12 days. **A** Immunostaining of iPSC-podocytes showing podocyte lineage markers PODXL (green), P-CADHERIN (green), MAFB (red), SYNPO (green), and F-actin (red) for cytoskeleton. Scale bar is 100 μm. **B** Pseudo-colored scanning electron microscopy image of an iPSC-podocyte in blue showing large cell bodies with foot-like projections and extracellular vesicles. Scale bar is 30 μm. **C** Transmission electron microscopy image of podocytes showing tight junction-like structures between cells (black arrows). Scale bar is 400 nm. **D** Flow cytometry histogram plots of the podocyte markers SYNPO and WT1 stained with a FITC and Texas Red secondary antibody (*n* = 3). **E** Quantitative analysis of podocyte markers such as PODXL, WT1, SYNPO, and NEPHRIN throughout the differentiation process was analyzed using (i) RT-PCR and (ii) Western blot quantification with β-actin as control. *Indicates *p* < 0.05

**Fig. 4:**
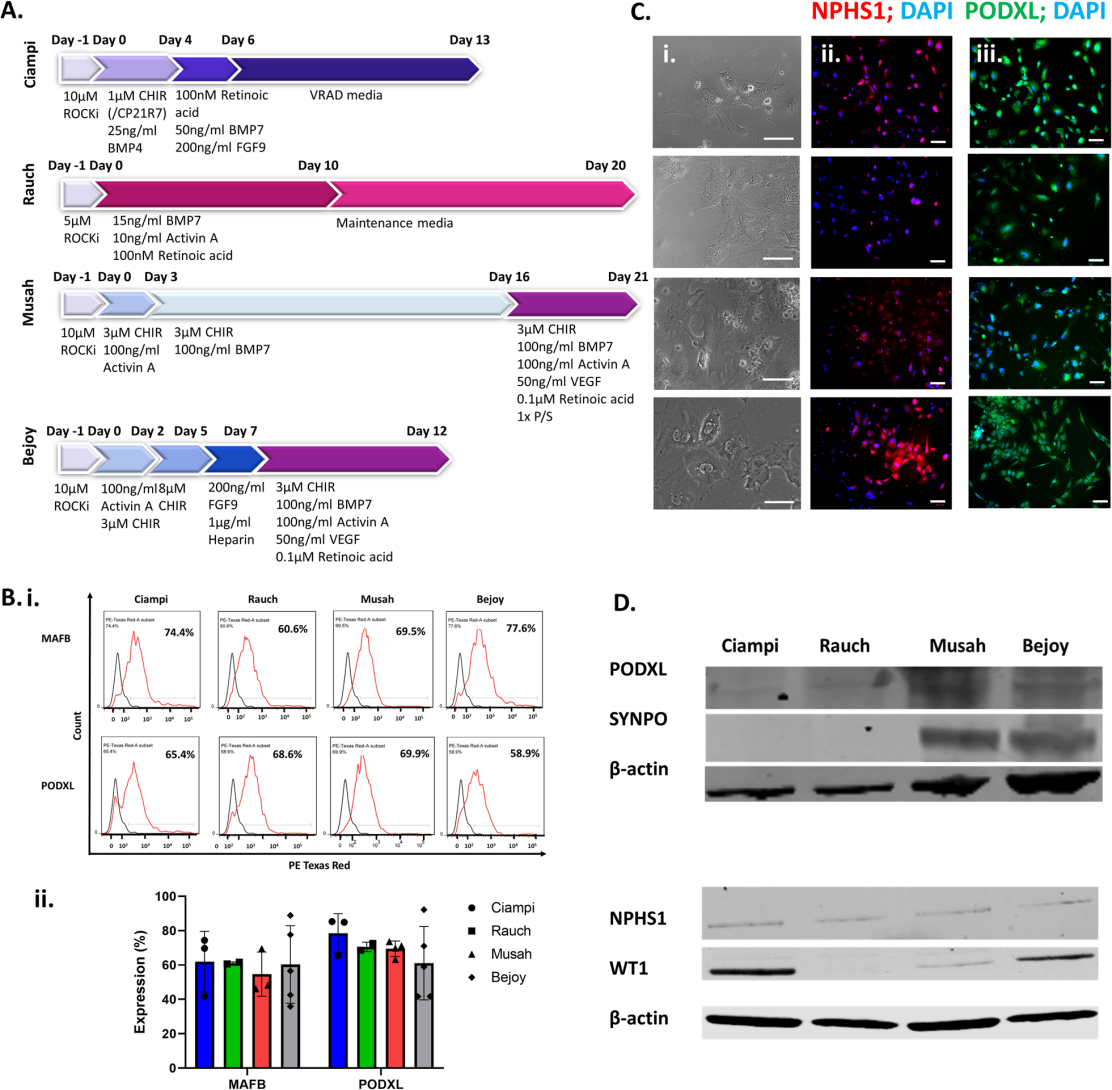
Comparison of the protocol with existing protocols. **A** Schematic of the podocyte differentiation protocols compared including key media components. **B**(i) Comparison of the podocyte markers MAFB and PODXL expression using flow cytometry analysis (*n* = 3). (ii) Quantification of (Bi). **C** Immunostaining of iPSC-podocytes with markers PODXL, NPHS1 in the compared protocols (*n* = 3). Scale bar 100 μm. **D** Western blot analysis of the podocyte markers PODXL, SYNPO, NPHS1, and WT1 as well as loading control β-actin in the compared protocols (*n* = 3)

Immunostaining of day 12 podocytes showed expression of podocyte markers musculoaponeurotic fibrosarcoma oncogene family B (MAFB), PODXL, SYNPO, NPHS1, and placental cadherin (P-cadherin; Figs. 3A, 4C). Fluorescent-labeled phalloidin was used to visualize F-actin cytoskeletal bundles in differentiated podocytes (Fig. 3A). We performed flow cytometry analysis and found that derived podocytes expressed Wilms’ tumor suppressor gene 1 (WT1) (89.7%), MAFB (77.6%), and PODXL (58.6%), indicating efficient production of podocytes from iPSCs (Figs. 3D, 4B). We also characterized the expression of podocyte-associated transcripts throughout the differentiation process by RT-PCR. We found that differentiated cells had increased levels of podocyte mRNA markers including *WT1*, *PODXL*, and *SYNPO* (Fig. 3Ei). We found increased expression of the podocyte marker SYNPO in the differentiated podocytes following quantification of marker protein levels by Western blot analysis as well (Fig. 3Eii). Immunostaining indicated that iPSC-derived podocytes produced the actin-associated protein SYNPO in a filamentous pattern. As protocols starting with iPSCs tend to produce more immature cell types, it was not surprising that expression of SYNPO, which is specific to postmitotic differentiated podocytes, was lower (44.5%) than other markers that are less specific to mature podocytes (Fig. 3A). Scanning electron microscopy (SEM) analysis of the resulting podocytes revealed thin foot processes extending from cells (Fig. 3B). Transmission electron microscopy (TEM) analysis at higher magnification revealed tight junctions formed between podocytes (Fig. 3C).

### Comparison of different methods of iPSC-podocyte differentiation

Next, we carefully compared four protocols for the differentiation of iPSC-derived podocytes derived from the DYR0100 iPSC line (Fig. 4). The differentiation efficiency of the accelerated protocol [14] was compared to Ciampi [17], Rauch [41], and Musah [12] protocols. Each protocol involves differentiation in different defined mediums, with major variation in the length of time required for each step and the composition of the various media (Fig. 4A). Our protocol begins and ends with the same media as described in the Musah protocol, but was shortened by adding defined steps to reach the nephron progenitor cell type through modulation of the Wnt pathway. Whereas in the Musah protocol the concentration of the Wnt activator and glycogen-synthase kinase-3 (GSK-3) inhibitor CHIR99021 was held constant [12], in our protocol CHIR99021 was increased from 3 to 8 μM for two days. Next, we withdrew CHIR99021 to stop Wnt activation, similar to the 13-day Ciampi protocol [17]. To induce the nephron progenitor cell type, cells were cultured for two days in media including FGF9 stabilized by heparin without Wnt activation. This period required 4 days, whereas the Musah protocol required 13 days (Fig. 4A). The other lengthy protocol, Rauch, does not rely on Wnt activation [41].

We employed immunofluorescence, flow cytometry analysis and Western blots to compare the expression of podocyte-specific proteins (Fig. 4B–D). Among the four protocols, Rauch had the lowest expression of each marker by every analysis technique (Fig. 4B–E). Ciampi had low expression of SYNPO and NPHS1 by Western blot, but PODXL staining by Western, immunofluorescence, and flow cytometry were high (Fig. 4B–E). Compared to the Musah protocol, we found our protocol produced similar levels of podocyte marker PODXL and elevated levels of NPHS1 by immunofluorescence, comparable levels of MAFB and PODXL by flow cytometry analysis, and comparable levels of PODXL, SYNPO, and NPHS1 with higher levels of WT1 by Western blot (Fig. 4B–D). We found all protocols had comparable numbers of cells that were MAFB + or PODXL + by flow cytometry analysis (Fig. 4Bi,ii). iPSC-derived podocytes differentiated by the Ciampi and Rauch protocols have podocyte specification, but the expression levels of podocyte markers by Western blot were lower than the Musah or Bejoy protocols (Fig. 4D). Therefore, our shorter protocol produced iPSC-derived podocytes in similar numbers and with a maturity level that is equal to or better than established methods of podocyte differentiation.

### iPSC-derived podocytes integrated into mouse embryonic kidney organoids

**Fig. 5:**
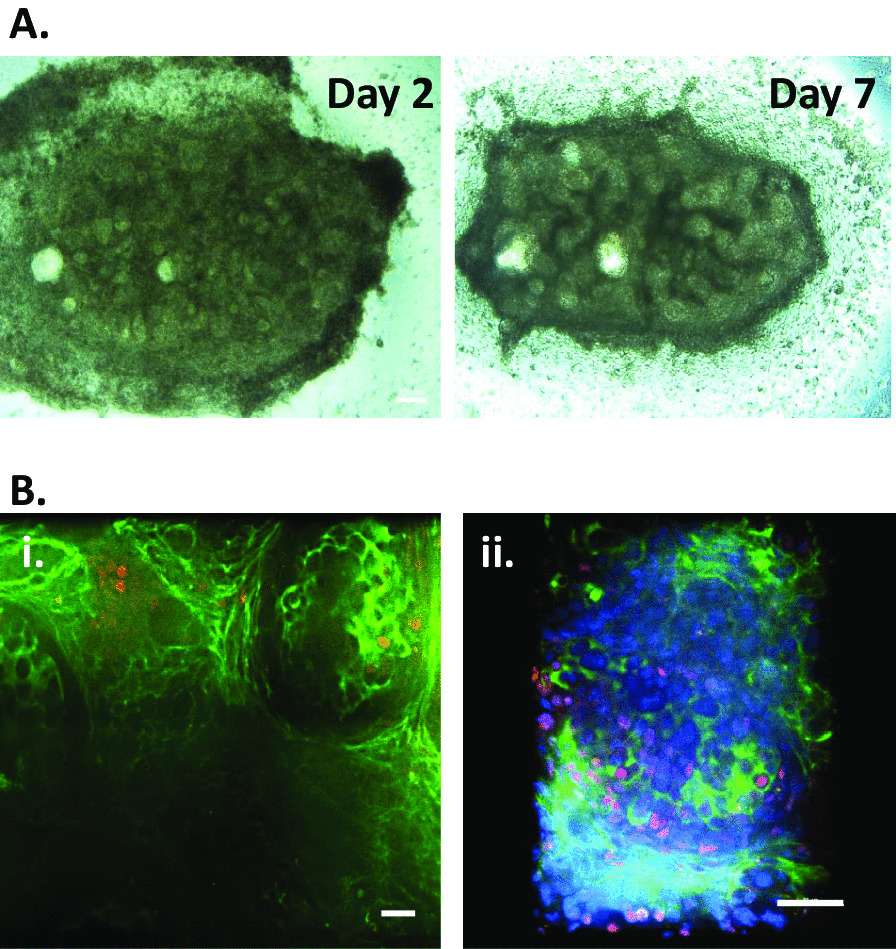
Integration of iPSC-derived podocytes into mouse embryonic kidneys. **A** Brightfield images of the recombinant organoids generated using iPSC-podocytes and E12.5 embryonic kidney at day 2 and day 7 after initiation of ex vivo co-culture. **B** Higher (40x, i) and lower (20x, ii) magnification images showing the iPSC-derived podocytes (human-specific MAFB+, red) incorporated into mouse glomerular structures (mouse-specific NPHS1+, green) (*n* = 2). Counterstaining with DAPI (blue). Scale bar is 50 μm

Ex vivo organoid culture is a widely accepted assay to verify the potential of cells to integrate into the expected cellular niche [16, 49, 50]. Previous reports showed that nephron progenitor cells can integrate into the endogenous nephron progenitor field and can proliferate within the assay [49, 50]. Therefore, the ability of iPSC-derived podocytes to integrate into the renal milieu was tested using cellular recombination [16]. Differentiated iPSC-podocytes were recombined with cells dissociated from mouse embryonic E12.5–E15.5 kidneys by centrifugation, and the resulting pellet was explant cultured for 7 days (Fig. 5A). The aggregate was fixed on day 7. Both a human-specific MAFB antibody and a mouse-specific NPHS1 antibody were used to track the podocytes in the hybrid glomeruli. Immunostaining revealed close interaction of the MAFB + iPSC-podocytes with mouse cells that were NPHS1 + within the glomeruli. MAFB + human podocytes were predominantly concentrated on the periphery of aggregate recombinations (Fig. 5Bi, ii).

### iPSC-derived podocyte: functional validation and modeling of diabetic kidney disease

The endocytic absorption of albumin is an approximate measure of the functionality of podocytes. FITC-albumin uptake by iPSC-derived podocytes was measured by imaging on a fluorescence microscope. iPSC-derived podocytes cultured for 24 h at 37 °C had uptake of FITC-albumin into the cytoplasm, whereas podocytes cultured at 4 °C failed to endocytose albumin [51] (Additional file 1: Fig. S3). This temperature-dependent uptake is consistent with data from immortalized podocyte cell lines [52].

**Fig. 6:**
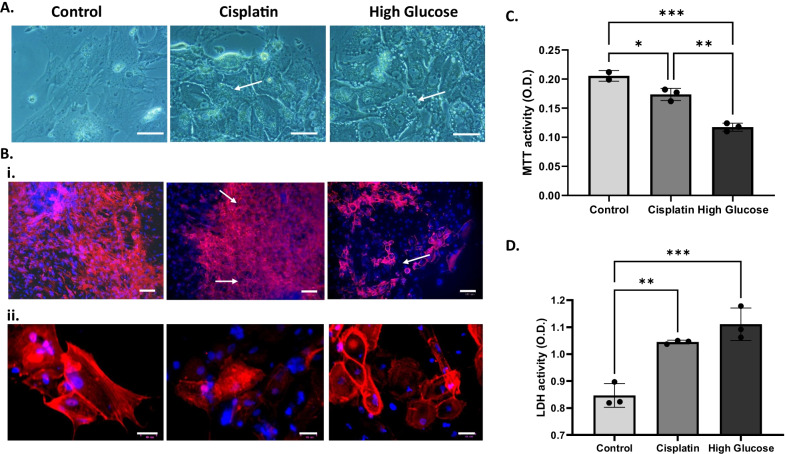
Modeling diabetic kidney disease by high glucose treatment of iPSC-derived podocytes. **A** Brightfield image of cisplatin- or high glucose-treated podocytes showing damage to the junctions between cells (arrows). Scale bar represents 50 μm. **B**(i) Reorganization of F-actin (phalloidin-red) along the cell periphery after the treatment with either Cisplatin (5 µM) or high glucose (100 mM). Scale bar is 100 μm. (ii) Higher magnification images showing out-of-order and intertwined actin fibers in treated groups compared to control groups podocytes with paralleled fasciculate models (*n* = 2). Scale bar is 30 μm. **C** Viability analysis by MTT assay showing reduction in podocyte viability after treatment with glucose. **D** Cytotoxicity analysis using LDH assay indicating increased toxicity within the podocytes treated with glucose. *Indicates *p* < 0.05

The differentiated podocytes were treated with 100 mM glucose for 48 h as a high glucose condition to model diabetic nephropathy. As a positive control for cell death, we treated podocytes with cisplatin. A nephrotoxic medication damages MAFB + cells [53]. Brightfield images revealed that cells treated with either cisplatin or high glucose had lost their cellular integrity (Fig. 6). Compared to control cells, F-actin staining demonstrated cytoskeletal remodeling within treated cells (Fig. 6B). Higher magnification photos revealed disordered, entangled actin fibers in treated groups, whereas control podocytes matched fasciculate models (Fig. 6B). Different biochemical assays were used to assess the damage caused by glucose to the podocytes. The high glucose-treated podocytes had lower viability than the untreated control group by MTT assay (Fig. 6C). To investigate the cytotoxicity, we performed an LDH assay and found that podocytes subjected to the high glucose condition had an increase in cytotoxicity (Fig. 6D). Together, these data indicate that iPSC-derived podocytes can be used to model glomerular disorders such as diabetic kidney disease.

## Discussion

Although human primary podocytes can be isolated, dedifferentiation of cells in tissue culture causes cells to lose their podocyte identity over time [54, 55]. In addition, the limited availability of adult kidney samples and eventual senescence of primary cells limits the utility of primary CD133+/CD24+/PODXL+ cells isolated from adult human kidney [56]. Although less cumbersome than primary cells to culture, conditionally immortalized podocytes also have limited utility for disease modeling due to poor expression of some podocyte markers [57]. Attempts to generate podocytes directly from iPSCs have resulted in immature podocytes with limited functionality [17, 41]. The production of more functional iPSC-derived podocytes with higher levels of markers indicating maturity requires a longer culture time and more expensive medium components [12]. We devised a four-step technique to generate functional podocytes from iPSCs via nephron progenitors [14]. Activation of activin signaling along with Wnt induced posterior primitive streak formation from iPSCs [58]. Therefore, we used a combination of activin A with a lower concentration of the GSK-3 inhibitor/Wnt activator CHIR99021 to generate the posterior primitive streak [14]. The primitive streak identity was confirmed by immunostaining for MIXL1 at day 2 (Fig. 2B; Additional file 1: Fig. S1B). To direct cells to the intermediate mesoderm [13, 15–17], high Wnt signaling activation was initiated from day 3 to day 5. For confirmation, we stained for the intermediate mesoderm marker Paired Box 8 (PAX8; Fig. 2C). By withdrawing Wnt activation and treating with FGF9 plus heparin for stabilization, we subsequently differentiated these intermediate mesoderm cells into nephron progenitors and confirmed their identity with the markers SIX2 and CITED1 (Fig. 2D).

To generate podocytes from nephron progenitor cells, the epithelialization of these cells must be stimulated. One of the most essential growth factors in the differentiation of podocytes is RA. RA triggers stem cells to lose their self-renewing properties and differentiate. RA therefore modulates podocyte gene expression to encourage nephron progenitors to differentiate into podocytes. BMP7 is a member of the BMP family, which belongs to the TGF superfamily, and is found in a variety of organs including podocyte precursor cells [59, 60]. Activation of BMP7 signaling at some point in the protocol appears to be required for differentiation of podocytes from iPSCs [17, 21, 22, 54]. Another molecule important for podocyte survival is VEGF [12]. In the Musah protocol as well as in this study, a combination of BMP7, CHIR99021, activin A, RA, VEGF induced the differentiation of podocytes from nephron progenitors [12].

The podocytes derived from our accelerated protocol had the typical arborized morphology, comprised of a main cell body with extending processes (Fig. 1B). The iPSC-derived podocytes expressed podocyte markers including SYNPO, NPHS1, MAFB, P-cadherin, and PODXL (Fig. 3A). We analyzed flow cytometry data and found that the number of cells differentiated by each of the four protocols were comparable, between 58 and 75% (Fig. 4B–D). Western blot analysis of the total protein levels showed highly variable expression between the four protocols, suggesting major differences in podocyte maturity among cells differentiated to a podocyte phenotype (Fig. 4D). The Rauch protocol had the lowest expression of protein markers, suggesting the poorest differentiation. Interestingly, withdrawal of Wnt activation was present in both our 12-day protocol and the Ciampi 13-day protocol, whereas the longer protocols did not manipulate Wnt activation levels during the protocol; they were either activated with steady CHIR amounts (Musah) or not activated (Rauch). It is possible that oscillation of the Wnt pathway through modulation of the levels of Wnt activation may accelerate differentiation [61]. Both our accelerated protocol and the Ciampi protocol produced iPSC-derived podocytes with increased WT1 expression. As WT1 interacts with the Wnt pathway, this may be a response to the CHIR withdrawal and reintroduction in both protocols. Future optimization of Wnt oscillation may be able to further accelerate iPSC-podocyte differentiation. The marker PODXL must be present in both mature and immature podocytes for normal glomerular function [62]. The protein levels of this marker were highest for the Musah protocol followed by our protocol, with low levels in iPSC-podocytes derived by the Ciampi and Rauch protocols. The mature podocyte marker SYNPO had high expression in cells derived by our accelerated protocol, with levels that were comparable to iPSC-podocytes derived via the Musah protocol. Both our protocol and the Musah protocol employed VEGF to mimic endothelial signaling and had the highest levels of podocyte marker proteins. Therefore, simulation of endothelial to podocyte cross talk through the addition of VEGF may be one of the keys to induction of a more mature podocyte phenotype. NPHS1 levels were low but detectable, and the levels were comparable for all protocols (Fig. 4D).

We tested the attraction of the differentiated iPSCs for their native niche by integrating them into a developing kidney structure in a recombination assay with mouse embryonic kidney cells (Fig. 5A). This assay demonstrated podocyte identity via incorporation of MAFB + iPSC-podocytes into NPHS1 + mouse glomeruli structures (Fig. 5B). The ability to internalize albumin is one of the important features of mature podocytes [63]. Therefore, we incubated our iPSC-derived podocytes with FITC-albumin at various temperatures and showed temperature-dependent endocytosis of albumin (Additional file 1: Fig. S3). With this functional validation in place, we chose to investigate the utility of the iPSC-derived podocytes for disease modeling. Current human diabetic kidney disease models rely upon human renal biopsy samples to generate podocytes in vitro. In vitro diabetic nephropathy has been modeled by treating immortalized human podocytes with high glucose [64, 65]. However, immortalization and lengthy periods in tissue culture change cell death pathways. In this study, we found that treating iPSC-derived podocytes with high glucose damaged the integrity of actin filaments and changed the morphology of the podocytes (Fig. 6). Glucose treatment both increased podocyte cytotoxicity and decreased their viability. Immunofluorescence staining with phalloidin showed extensive reorganization of F-actin along the cell periphery following the treatment with high glucose, suggesting severe podocyte dysfunction (Fig. 6).

## Conclusions

Our accelerated method was able to produce podocytes that were comparable to existing methods by multiple markers. We have found a faster and lower-cost method to generate podocytes from iPSCs that was based on mimicking the developmental stages of the embryonic kidney. The immunostaining of the podocytes showed positive staining for podocyte markers including PODXL, NPHS1, SYNPO, and MAFB. Functionality was verified by endocytosis of FITC-albumin. Furthermore, a recombination assay of our iPSC-derived podocytes with renal cells from E12.5 mouse embryos showed integration of iPSC-derived MAFB + podocytes into NPHS1 + mouse glomeruli structures. Treatment of the derived podocytes with high glucose resulted in both actin rearrangement and cell death, suggesting the efficacy of these cells in modeling diabetic nephropathy. Our understanding of both genetic and environmental podocyte diseases relies upon human tissue culture models that are both practical and faithful to phenotypes found in vivo. Using this method, the ability to generate patient-specific podocytes will provide a new resource, with future applications in drug screening and genome editing.

## Supplementary Information

**Additional file 1.** Supplementary methods and supplementary figures 1–3.

## Data Availability

All data generated or analyzed during this study are included in this published article [and its supplementary information files].

## Abbreviations

APEL: Albumin polyvinyl alcohol essential lipids
BMP: Bone morphogenetic protein
C: Centigrade
cDNA: Complementary DNA
CHIR: CHIR99021; small molecule Wnt activator
cm^2^: Square centimeter
CITED1: Cbp/p300 interacting transactivator with Glu/Asp rich carboxy-terminal domain 1
*C*_t_: Cycle threshold
DAPI: 4′,6′-Diamidino-2-phenylindole; stains double-stranded DNA
DMEM/F12: Dulbecco’s modified eagle medium/nutrient mixture F12
DNA: Deoxyribonucleic acid
F-actin: Filamentous-actin
FBS: Fetal bovine serum
FGF9: Fibroblast growth factor 9
FITC: Fluorescein isothiocyanate
GAPDH: Glyceraldehyde-3-phosphate dehydrogenase
GFB: Glomerular filtration barrier
GSK-3: Glycogen-synthase kinase-3
h: Hour
IgG: Immunoglobulin G
iPSCs: Induced pluripotent stem cells
MAFB: Musculoaponeurotic fibrosarcoma oncogene family B
min: Minutes
MIXL1: Mix paired-like homeobox; primitive streak marker
ml: Milliliter
mTesR: A defined media for embryonic or induced pluripotent stem cell culture
N2: Neuro-2 medium supplement
NEAA: Non-essential amino acids
ng: Nanogram
nM: Nanomolar
NPHS1: Nephrin
NPHS2: Podocin
OCT-4: Octamer-binding transcription factor 4; pluripotency marker
P-cadherin: Placental cadherin
P/S: Penicillin/streptomycin
PAX8: Paired Box 8, intermediate mesoderm marker
PBS: Phosphate-buffered saline
PFA: Paraformaldehyde
PODXL: Podocalyxin
RA: Retinoic acid
RIPA: Radioimmunoprecipitation assay
RNA: Ribonucleic acid
ROCKi: Rho-associated kinase inhibitor Y-27632 dihydrochloride
RT: Room temperature
RT-PCR: Real-time reverse transcription-polymerase chain reaction
s: Seconds
SEM: Scanning electron microscopy
SIX2: SIX Homeobox 2
Stage I medium: Mesoderm differentiation medium
Stage II medium: Intermediate mesoderm induction medium
SYNPO: Synaptopodin
TEM: Transmission electron microscopy
TBS: Tris-buffered saline
TBST: Tris-buffered saline + 0.1% Tween 20
TOBB: Tris-buffered saline-based Odyssey Blocking Buffer
TOBBT: Tris-buffered Saline-based Odyssey Blocking Buffer + 0.2% Tween 20
μM: Micromolar
VEGF: Vascular endothelial growth factor
VRAD: Podocyte-maintaining medium containing Vitamin D3 and retinoic acid
Wnt: Wingless-related integration site
WT1: Wilms’ tumor suppressor gene 1
ZO-1: Zonula occludens-1
