# Supplementary material for: Podocytes derived from human induced pluripotent stem cells: characterization, comparison, and modeling of diabetic kidney disease
Source: Stem Cell Res Ther. 2022 Jul 26;13:355. doi: 10.1186/s13287-022-03040-6 (PMC9327311; doi:10.1186/s13287-022-03040-6)
Supplement: Supplementary file 1 — Additional file 1. Supplementary methods and supplementary figures 1–3. [file 13287_2022_3040_MOESM1_ESM.docx]

**Supplementary Information**

**Supplementary Methods**

**Albumin uptake assay**

The functionality of derived podocytes was measured using an albumin uptake assay (19; 23). Briefly, differentiated podocyte cultures were cultured in serum-free media for 24 h. The next day, cells were rinsed with PBS and incubated with 50 μg/ml FITC-conjugated bovine serum albumin (Thermo Scientific). For the albumin binding assay, the cells were incubated for 1 h at 4°C. To evaluate binding and endocytosis, cells were kept at 37°C for 24 h. Brightfield images and FITC images were taken using a ZOE fluorescence microscope (Bio-Rad Laboratories) and merging of the images were used to determine the albumin uptake.

**
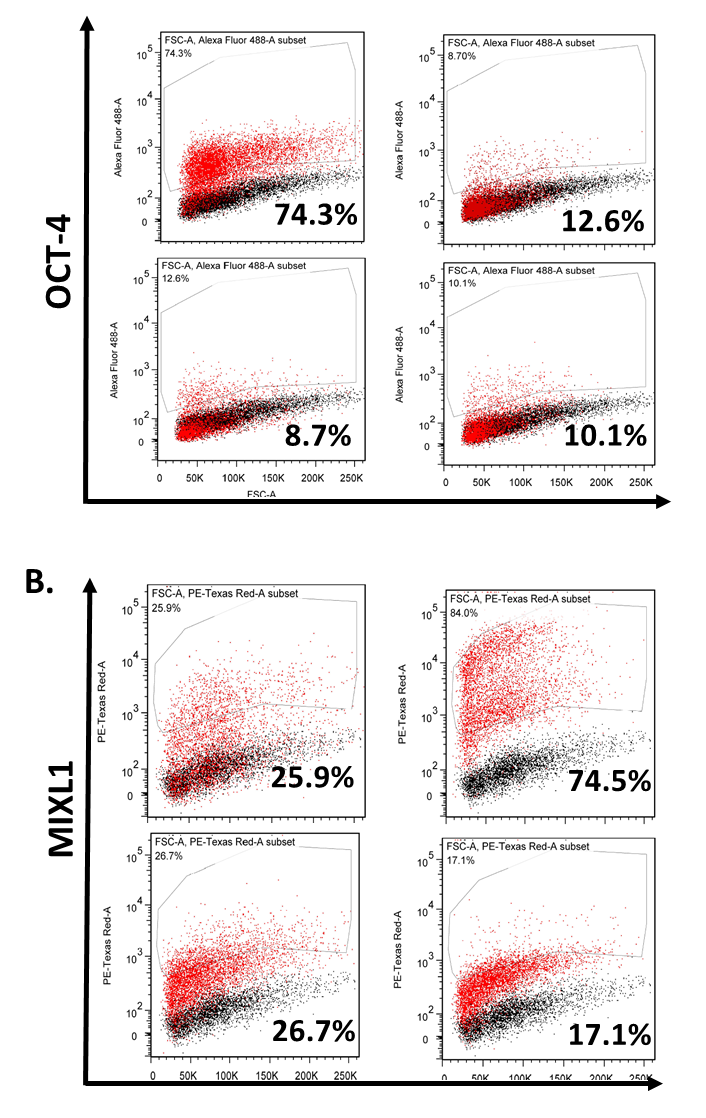
A.**

**Supplementary Figure 1. Characterization by flow cytometry of pluripotency and primitive streak markers at each stage of hiPSC-podocyte differentiation.** (A) Flow cytometry analysis of pluripotency marker OCT-4 at each stage of differentiation. First panel is identical to Figure 2E as the samples are from the same experiment. (B) Flow cytometry analysis of primitive streak marker MIXL1 expression at each stage of differentiation. Markers are plotted on the y-axis; forward scatter (FSC) is plotted on the x-axis.

**
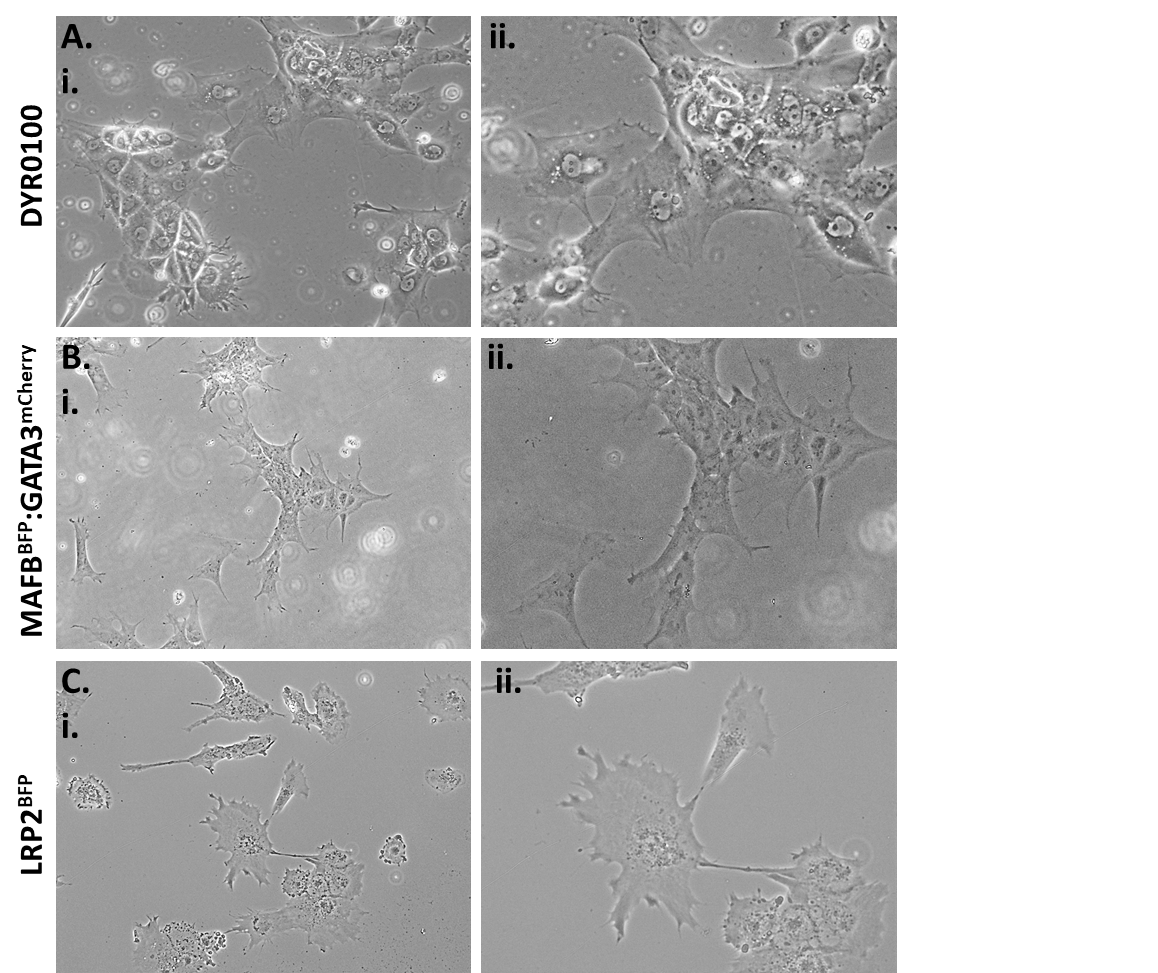
**

**Supplementary Figure 2. Characterization of podocyte cells derived from three iPSC cell lines.** Lower (i) and higher (ii) magnification brightfield images of the day 12 podocytes derived from the (A) DYR0100 iPSC cell line; (B) MAFB:mTagBFP2/GATA3:mCherry iPSC cell line; and (C) LRP2BFP:mTagBFP2 cell line.

**
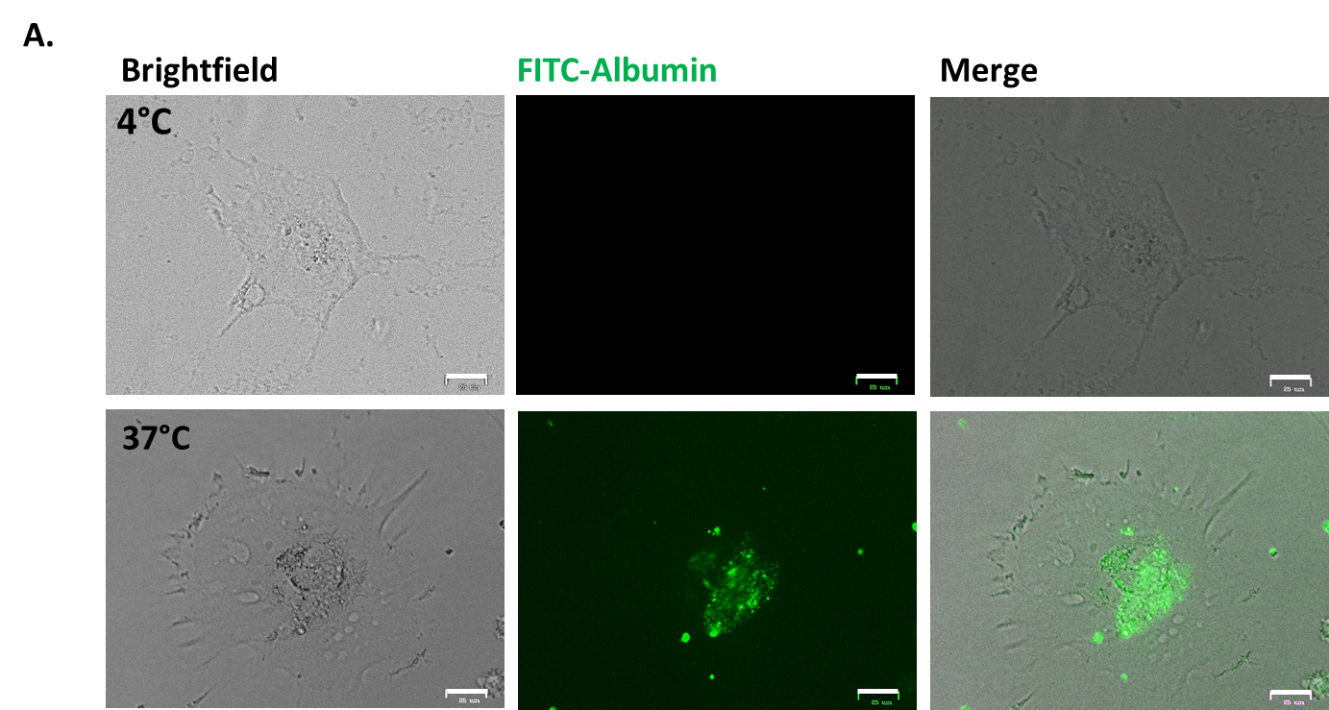
**

**Supplementary Figure 3. Functional validation of iPSC-derived podocytes.** iPSC-derived podocytes were incubated with FITC-albumin either for 1 h at 4°C or 24 h at 37°C showing endocytosis of the labeled albumin only at 37°C. Scale bar is 100 μm.
